# Expression profiling of suppressive monocytes (CD14+HLA-DRlow/neg) in cancer patients

**DOI:** 10.1186/2051-1426-1-S1-P142

**Published:** 2013-11-07

**Authors:** Stefan F Cordes, Michael P Gustafson, Zhe Zhang, Peggy A Bulur, Dennis A Gastineau, Yi Lin, Allan B Dietz

**Affiliations:** 1 Internal Medicine, Mayo Clinic, Rochester, MN, USA; 2 Transfusion Medicine, Mayo Clinic, Rochester, MN, USA; 3 Hematology, Mayo Clinic, Rochester, MN, USA; 4 Experimental Pathology, Mayo Clinic, Rochester, MN, USA

## Background

Immune evasion by suppression of host immunity is one of the major mechanisms that tumors use to promote its own survival. We have reported an abundance of immunosuppressive monocytes (CD14+HLA-DRlow/neg) in lymphoma (LYM), multiple myeloma (MM), glioblastoma multiforme (GBM), prostate cancer and renal cell carcinoma (RCC). The number of these cells increases with more aggressive disease and is associated with decreased survival. In this study, we examine the RNA expression profile of monocytes from cancer patients compared to healthy donors (CNTRL) to identify differentially affected functional pathways.

## Methods

Monocytes were isolated from peripheral blood using anti-CD14 immunomagnetic beads. RNA was extracted from the monocytes and analyzed via whole genome expression array (Affymetrix GeneChip Human Genome U133 Plus 2.0 arrays). The number and frequency of the HLA-DRlow/neg phenotypes were quantified, but the population was not purified. Data was analyzed using packages assembled from R and Bioconductor. Probe-level data from the GeneChips was background-corrected, normalized and summarized into a set of expression measures via the Robust Multi-array Average with GC-content background (GCRMA) method. Several search strategies were employed to find interesting gene expression differences: (1) Directed searches for specific genes, (2) Hypergeometric testing for Gene Set Enrichment Analysis and (3) Gene Set Enrichment Analysis using liner models. A variety of gene set was employed: (A) Gene Sets derived from the Gene Ontology (GO) database, (B) Gene Sets derived from the Kyoto Encyclopedia of Genes and Genomics (KEGG) database and (C) Gene Sets curated by the Broad Institute.

## Results and conclusions

Monocytes RNA expression profiles were obtained from 5 CNTRL, 5 LYM, 6 MM, 4 GBM, 5 RCC, 1 ovarian cancer. Comparison of the differences in monocyte RNA expression in cancer patients to normal controls found statistically highly significant under-expression of immune function genes such as those in the TLR and TNFaR superfamily and overexpression of genes regulating cell metabolism. These genes lie at the intersection of several oncogenic and immunogenic gene sets and their roles in immunosuppressive monocytes are under active investigation. Gene Set Enrichment analysis of gene expression profiles is a promising exploratory avenue towards identifying novel immuno-regulatory pathways of CD14+HLA-DRneg/low monocytes.

